# Aptamers, Riboswitches, and Ribozymes in *S. cerevisiae* Synthetic Biology

**DOI:** 10.3390/life11030248

**Published:** 2021-03-17

**Authors:** Huanhuan Ge, Mario Andrea Marchisio

**Affiliations:** School of Pharmaceutical Science and Technology, Tianjin University, 92 Weijin Road, Tianjin 300072, China; gehuanhuan@tju.edu.cn

**Keywords:** *S. cerevisiae*, aptamers, riboswitches, ribozymes, synthetic biology

## Abstract

Among noncoding RNA sequences, riboswitches and ribozymes have attracted the attention of the synthetic biology community as circuit components for translation regulation. When fused to aptamer sequences, ribozymes and riboswitches are enabled to interact with chemicals. Therefore, protein synthesis can be controlled at the mRNA level without the need for transcription factors. Potentially, the use of chemical-responsive ribozymes/riboswitches would drastically simplify the design of genetic circuits. In this review, we describe synthetic RNA structures that have been used so far in the yeast *Saccharomyces cerevisiae*. We present their interaction mode with different chemicals (e.g., theophylline and antibiotics) or proteins (such as the RNase III) and their recent employment into clustered regularly interspaced short palindromic repeats–CRISPR-associated protein 9 (CRISPR-Cas) systems. Particular attention is paid, throughout the whole paper, to their usage and performance into synthetic gene circuits.

## 1. Introduction 

RNA is a polymeric biomacromolecule that carries out crucial functions in diverse biological systems. Since it was discovered, the messenger RNA (mRNA) has shown its fundamental role in gene expression as the intermediate step from DNA to proteins in the central dogma. Further, noncoding RNA elements are ubiquitous both in prokaryotic and eukaryotic cells. Over the last two decades, with the advent of synthetic biology, noncoding RNAs have been largely used to control protein synthesis within genetic circuits [1]. To this aim, riboswitches and ribozymes have proved to be powerful solutions due to their capability to interact with chemicals, i.e., circuit inputs [2].

Riboswitches are regions of the mRNA that contain specific evolutionarily conserved ligand-binding domains (sensors) along with a variable sequence, termed the expression platform, which enables regulation of the downstream coding sequence (CDS) [3]. They were discovered in 2002 in bacteria [4,5,6] and later exploited in numerous species for tuning RNA stability [7], transcription [8], translation [9], and RNA splicing [10]. Riboswitch sensors are RNA aptamers, i.e., hairpin structures that bind small molecules with high affinity and specificity. Usually, synthetic aptamers are produced through in vitro or in vivo selection—systematic evolution of ligands by exponential enrichment (SELEX) [11,12]. Artificial aptamers have been selected to bind different bio-targets such as dyes, proteins, aromatic small molecules, and antibiotics [13]. Upon ligand binding, aptamers undergo conformational changes that have an impact on the tertiary structure (folding) of the mRNA chain where the riboswitch is included. This explains why natural and synthetic riboswitches are a means of controlling translation initiation.

Ribozymes—whose existence have been known since the 1980s of the last century [14]—are functional RNA molecules able to catalyze biochemical reactions such as the cleavage and the ligation of phosphodiester and peptide bonds [15,16,17]. Therefore, in their activity they resemble enzyme proteins. However, ribozymes have unique characteristics and advantages over proteinic enzymes: (1) They are functional RNA transcripts, i.e., they do not basically demand any particular post-transcriptional modifications; (2) the expression level of functional RNA transcripts is more controllable and predictable than that of proteins because of the absence of the translational step; (3) transcription consumes less energy and resources than translation; (4) manipulation of ribozyme functionality is relatively easy compared to that of polypeptides; and (5) the activity and the stability of ribozymes can be tuned via simple modifications [18]. The catalytic activities of ribozymes are generally categorized into three groups: Cleavage, splicing, and other functions [18]. Cleavage is the most widespread task among ribozymes. We find it in self-cleaving hammerhead ribozymes (HHR) [19], simple hairpins [20], Varkud satellites (VS) [21], hepatitis delta virus (HDV) ribozymes [22], glucosamine-6-phosphate synthase (glmS) ribozymes [23], and twister ribozymes [24]. Aptamers fused to natural, self-catalytic RNA structures are, in general, referred to as aptazymes [25,26]. They have broadened the potential of riboswitches/ribozymes for predictable gene regulation and have been widely engineered [27].

In this paper, we reviewed RNA structures such as aptamers, ribozymes, and riboswitches that have found synthetic biology application in the yeast *Saccharomyces cerevisiae*. We first described aptamers that react to different kinds of commonly used chemicals. Then, we presented how ribozymes and other hairpin structures have been utilized together with clustered regularly interspaced short palindromic repeats–CRISPR-associated protein 9 (CRISPR-Cas) systems. We illustrated switches arising from hairpins that are cleaved by RNase III, and, finally, before the conclusions, we listed some other RNA structures that do not belong to any specific groups.

## 2. Ligand-Responsive Aptamers in *S. cerevisiae*

Most of the ribozymes and riboswitches that have shown high functionality in *S. cerevisiae* respond to well-tolerated drugs, such as theophylline, tetracycline, and aminoglycosides. They are thoroughly described in the following. Examples of other RNA structures sensing different chemicals are given as well, at the end of this section.

### 2.1. Theophylline Aptamers

**Figure 1:**
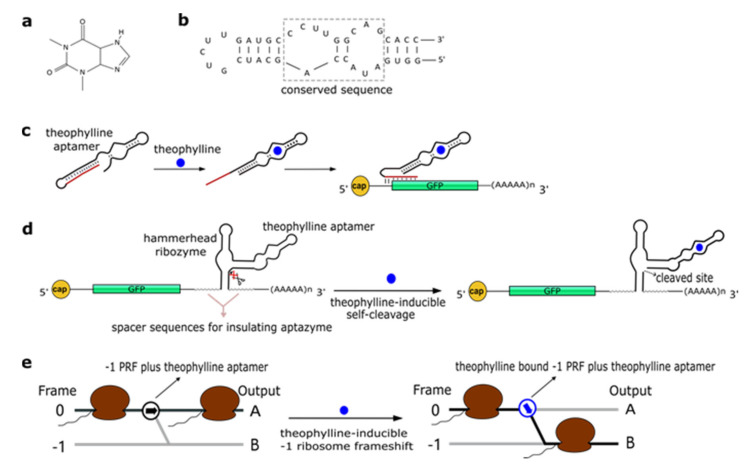
The theophylline aptamer. (**a**) Theophylline structure (PubChem CID 2153). (**b**) The secondary structure of the theophylline aptamer. (**c**) The theophylline-responsive antiswitch as a means to control gene expression [32]. The RNA sequence complementary to the target green fluorescence protein (GFP) transcript is highlighted in red. (**d**) Fusion of a theophylline aptamer to the stem II of a hammerhead ribozyme. The resulting aptazyme undergoes autocleavage in the presence of 5 µM of theophylline causing an over 15-fold decrease in fluorescence expression. (**e**) Theophylline-induced translational frame shift. Here, the theophylline aptamer is fused to a ‒1 programmed ribosomal frameshifting (PRF) RNA structure [37].

Theophylline (1,3-dimethylxanthine—see Figure 1a), a common medicine for the treatment of chronic asthma, is a natural product extracted from the leaves of plants such as *Camellia sinensis* (tea plant) and *Theobroma cacao*. In 1994, Jenison et al. [28] used SELEX to isolate RNA molecules with high affinity for theophylline. From the pool of selected molecules, TCT8-4 RNA was truncated into the mTCT8-4 aptamer that is now simply referred to as the canonical theophylline aptamer [29]. This sequence shortening increased the affinity of the RNA for the drug, as the dissociation constant K_D_ decreased from 0.6 to 0.1 µM [28]. The theophylline aptamer (Figure 1b) is over 30-nt long and includes a core made of fifteen conserved residues, organized into two internal loops, which is required for theophylline binding [28,30,31]. In particular, C27 nucleotide is the key residue in recognizing theophylline and discriminating against caffeine [31]. The aptamer manifests a structural change upon chemical binding (conformational capture, or selection and mechanism). Initially, this RNA structure was utilized to build allosteric ribozymes such as the above-mentioned aptazymes. In 2005, Bayer and Smolke [32] developed a class of small, modular trans-acting RNAs, termed antiswitches, which regulated gene expression in a tunable ligand-dependent manner. They fused theophylline (mainly) and tetracycline aptamers to an antisense RNA sequence that was designed to match a fifteen-nucleotide region near the start codon of the target mRNA (encoding for the green fluorescence protein—GFP) (Figure 1c). Implementation of antiswitches in *S. cerevisiae* determined a sharp reduction (OFF switch) or increase (ON switch)—about 90% in both cases—in GFP expression in the presence of 1 to 10 mM of theophylline. This work represented the first successful application of theophylline-dependent riboswitches in eukaryotic cells.

In a later work, Win and Smolke [33] modified an HHR with the insertion of either a theophylline or a tetracycline aptamer at the stem II by means of a communication module (referred to as transmitter). Chemical binding to the aptamer triggered the tertiary interactions between loop I and II, necessary either to induce or block self-cleavage. Stem III was used to integrate the synthetic ribozyme in the 3′UTR (untranslated region) of the target mRNA (Figure 1d). This “RNA-based gene-regulatory platform” showed, in *S. cerevisiae*, its utility in the control of cell growth and the detection of metabolites such as xanthine. This design was subsequently modified with the usage of a transmitter to link an aptamer to either stem I/stem II, or another aptamer. Single-input complex devices and two-input Boolean gates (such as AND, NAND, NOR, and OR) sensing theophylline (5–10 mM) and/or tetracycline (0.25–0.5 mM) were engineered by means of two modified HHRs or a single one carrying an aptamer at both stem I and stem II or two aptamers in series at stem II. A remarkable ~14-fold gain in fluorescence expression was achieved by the AND gate [34]. A single HHR modified with the addition of only one theophylline aptamer (tetracycline in few variants) was then used to establish a two-color fluorescence-activated cell sorting (FACS)-based screening approach [35]. This modular design for theophylline-responsive ribozymes was, furthermore, adopted to implement a novel screening system for enzyme activity. By exploiting the fact that theophylline is produced via caffeine demethylation, the activity of a caffeine demethylase in *S. cerevisiae* could be assessed by the fluorescence level triggered by an ON switch sensing the amount of theophylline (up to 140 µM) derived from (up to 1 mM) caffeine transformation. This screening process permitted us to find out mutations that increased, considerably, both the activity and the selectivity of caffeine demethylase [36].

A peculiar implementation of RNA-based Boolean gates and an apoptosis controller was presented by Anzalone et al. [37] who engineered ligand-responsive ‒1 programmed ribosomal frameshifting (PRF) elements by coupling them with either theophylline or neomycin aptamers. Eukaryotic ‒1 PRF signals, present inside mRNA transcripts, are made of two main features: (1) The slippery site, where the frameshift takes place, which corresponds to two homopolymeric triplets characterized by the sequence XXXYYYZ (X denoting any nucleotides; Y standing for A or U; and Z meaning A, C or U); and (2) a stimulatory RNA structure (a hairpin or a pseudoknot) located downstream of the heptanucleotide. When, during the elongation phase, ribosomes meet a ‒1 PRF signal along the mRNA, a fraction of them slip back by a single nucleotide, shifting translation to the ‒1 reading frame, with a consequent change in the polypeptide downstream of the ‒1 PRF signal (Figure 1e) [38]. Anzalone and co-authors combined optimized ‒1 PRF RNA structures with aptamers either to prevent (OFF switch) or enhance (ON switch) the frame shift in translation in the presence of the aptamer-binding ligands. In both configurations, theophylline aptamers slightly outperformed neomycin aptamers. In particular, 40 mM theophylline led either to a 7.0-fold increase or a 5.9-fold decrease of the translation frame shift, whereas 550 µM neomycin allowed only either a 5.0-fold enhancement or a 4.2-fold reduction of the same event. Two OFF switches led to a NOR gate implementation, whereas an AND gate demanded two ON switches. A theophylline ON switch coupled to a neomycin OFF switch was, in contrast, at the basis of the apoptosis controller. Each synthetic circuit was shown to work with high efficiency, with the apoptosis controller allowed us to register an over 300-fold decrease in cell viability in the presence of both inputs.

### 2.2. Tetracycline Aptamers

**Figure 2:**
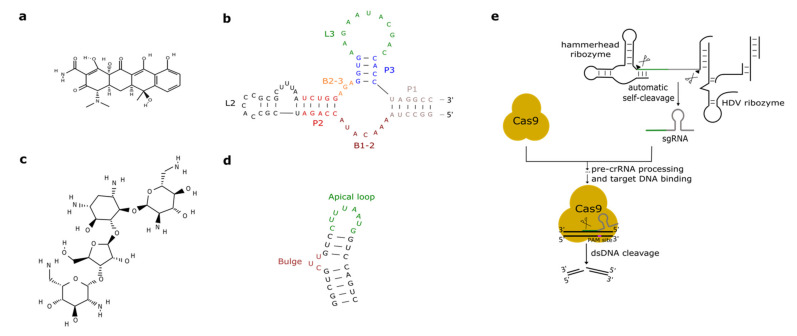
Antibiotic-responsive aptamers and the ribozyme-gRNA-ribozyme (RGR) cassette. (**a**) Tetracycline structure (PubChem CID 54675776). (**b**) The tetracycline aptamer. (**c**) Neomycin structure (PubChem CID 8378). (**d**) The neomycin aptamer. The main structural features of the two RNA molecules are here highlighted. (**e**) RGR cassette for the expression of single guide RNAs. Upon autocleavage of the HH and the hepatitis delta virus (HDV) ribozymes, the single guide RNA is released. sgRNA binds Cas9 and brings it to the target DNA that is finally cut. The green line denotes the spacer, whereas the gray hairpin represents the direct repeat.

Promoters activated or repressed by tetracycline (tc—see Figure 2a) have been used in yeast bio-engineering for over twenty years [39,40] since tc is an easy-to-handle antibiotic that does not interfere severely with yeast cellular metabolism [41].

As we have seen in the previous section, the tc aptamer has been used in several works together with the theophylline aptamer. The tetracycline aptamer consists of three stems (P1–P3), two single-stranded regions (the bulges, B1-2 and B2-3), and the loops L2 and L3 (see Figure 2b). Portions of both bulges and the L3 loop are involved in tc binding [42] that takes place at a very high affinity (K_D_ equals 0.8 nM [43]). In 2003, Suess et al. [44] reported the application of a tetracycline-binding aptamer to the control of gene expression in *S. cerevisiae*. Green fluorescence was measured after placing the tc-responsive riboswitch in the 5′UTR of the strong *ADH1* promoter (pADH1), either close to the fluorescent protein start codon or in a cap-proximal location. The latter position appeared more effective in repression of translation (6-fold in the presence of tetracycline). Interestingly, stabilization of the aptamer structure led to an increased ON-to-OFF ratio but lowered basal fluorescence expression (i.e., in the absence of tetracycline). In another work published in 2003, the tetracycline riboswitch was shown to lead, in the presence of the antibiotic, to a 9-fold downregulation in luciferase synthesis from the constitutive *TEF1* promoter [45]. 5′UTRs containing a variable number (one to three) of tetracycline aptamers were analyzed in [46]. Initially, each configuration was placed between the *ADH1* promoter and the *GFP* gene. As already experienced in [44,45], riboswitches lowered the cell fluorescence level considerably when already in the absence of tetracycline. However, in the presence of 250 µM of the chemical, two and three aptamers caused an almost complete loss of fluorescence (1.1% and 0.6% of the fluorescence produced by pADH1 without aptamers) with a 21-fold and 37-fold repression factor compared to tetracycline-untreated cells. These riboswitch-containing 5′UTRs, preceded by either pADH1 or the *GPD* promoter, were then adopted to construct universal insertion cassettes that could be integrated, via homologous recombination, into the yeast genome. Five endogenous genes (NEP1, NOP8, NOP14, PGI1, and SEC1) were completely switched OFF upon induction with at most 500 µM of tetracycline. NOP8 and SEC1 had never been regulated previously. More recently, the tandem configuration of this riboswitch was optimized in silico via machine learning techniques. In vivo, the newly designed 5′UTR sequence guaranteed a 40-fold repression of fluorescence in the presence of 250 µM tetracycline [42].

One or two tetracycline-responsive riboswitches were also shown to be effective in controlling pre-mRNA splicing when placed within an intron nearby the 5′ splice site (SS). The most efficient regulation was obtained by burying the 5′ SS into the P1 stem at the basis of the tetracycline aptamer. Upon insertion of the modified intron along the sequence of GFP, fluorescence was emitted by the cells only in the absence of tetracycline, i.e., when the mRNA was spliced successfully. In contrast, in the presence of tetracycline a drastic reduction in cell fluorescence level was observed [47].

In a different approach to control gene expression, the tetracycline aptamer was fused to the full-length HHR N79 from *Schistosoma mansoni* via a linker region optimized through 11 rounds of in vitro selection (SELEX). Synthetic ribozymes completely self-cleaved in the presence of 1 µM of tetracycline, whereas self-cleavage was inhibited up to 333-fold in the absence of the antibiotic [48].

### 2.3. Aminoglycosides-Responsive Aptamers

Aminoglycosides are positively charged antibiotics characterized by a high affinity towards RNA sequences [49,50,51]. Among them in the 1990s, neomycin was shown to have a potent inhibitory action on different kinds of ribozymes [52,53]. Hence, Weigand et al. [54] employed neomycin (Figure 2c) to develop a two-stage method—in vitro selection followed by in vivo (yeast) screening—to identify novel ligand-binding riboswitches. With this procedure, several new artificial aptamers responding to neomycin were engineered. The most performant one, termed M4, induced a 7.5-fold fluorescence repression in the presence of 100 µM neomycin. Another neomycin-binding aptamer, N1 (see Figure 2d), was subsequently deeply studied and its interactions with different aminoglycosides (such as ribostamycin, tobramycin, and paromomycin) were clarified [55,56,57,58,59].

Klauser et al. [60,61] introduced a new design for synthetic ligand-responsive ribozymes. The main idea was to preserve, as far as possible, the structure of a native HHR to which either a theophylline or a neomycin (N1) aptamer was attached. This was achieved by placing the aptamer at the 5′ end (stem III) of a type 3 *S. mansoni* HHR. Neomycin-responsive ribozymes, engineered in this way, were inserted in the 3′UTR of the *gal4* gene. Hence, *gal4* expression was maximal in the absence of neomycin. The Gal4 activator induced the synthesis of *lacZ* such that the ribozyme performance was assessed, indirectly, via beta-galactosidase assay. This represented a new protocol for the in vivo selection of neomycin-sensing ribozymes. The best one reported in this work showed a ~25-fold reporter downregulation in the presence of 100 µg/mL neomycin. A similar design was followed, in a later work by the same group [62], for the engineering of a neomycin-responsive riboswitch, based on an inactive env-9 twister ribozyme, which produced a ~10-fold downregulation in the *lacZ* gene expression.

In a recent work, Sack et al. [63] presented a new design for the neomycin ribozyme where a neomycin aptamer (M4 or M7 [54]) was placed at stem I of the *S. mansoni* HHR. The connection between aptamer and stem I was realized through an addressable three-way junction [64], whose flexibility was increased with the addition of a three-nucleotide bulge (UAU). The synthetic ribozyme was inserted in the 3′UTR of the *gfp* gene such that its activity was quantified, directly, by measuring cell fluorescence. Libraries of artificial ribozymes, obtained by randomizing, first, the three-way junction and, then, the three-nucleotide bulge, were screened in the presence and absence of, usually, 100 µg/mL of neomycin. Differently from [65], both OFF and ON switches were found, i.e., the binding of neomycin, in some cases, made the HHR fold into an inactive conformation and cause an increase in green protein synthesis. ON switches achieved a ~2-fold fluorescence increase, whereas OFF switches caused a ~3-fold decrease in fluorescence.

A new synthetic riboswitch responding to paromomycin [66] was engineered after screening a library of 10^15^ RNA sequences via the Capture-SELEX method [67]. With respect to SELEX, Capture-SELEX allows, in vitro, a first selection of aptamers that undergo structural modification upon binding the chosen ligand. This lowers considerably the number of riboswitches (or ribozymes) to be tested, subsequently, for their in vivo functionality. The synthetic PARO riboswitch is 44-nt long and consists of two hairpins (P1 and P2) with the docking site located on P2. It binds paromomycin with high affinity (K_D_ = 20 nM) and allows an 8.5-fold translation downregulation to be registered when exposed to 250 µM of the antibiotic. On it, a working NOR gate was engineered by replacing P1 with the neomycin aptamer. In the presence of 250 µM of both inputs, the logic gate gave a 13.8-fold repression factor.

### 2.4. Other Aptamers Used in S. cerevisiae

As we have seen above, antibiotics such as tetracycline and aminoglycosides have been largely used for the engineering of novel aptamers. Groher et al. [68] realized a synthetic riboswitch responding to a new kind of antibiotic, the fluoroquinolone ciprofloxacin. Tested on GFP expression, the riboswitch showed a 7.5-fold fluorescence downregulation and a good affinity towards its ligand (K_D_ = 64.2 nM).

In 2001, Grate and Wilson [69] described a method to influence *S. cerevisiae* cell cycle via a dye-responsive aptamer placed in the 5′UTR of the *CLB2* gene. Upon binding tetramethylrosamine (a malachite green analogue), the aptamer underwent a drastic structural change that reduced *CLB2* translation in a considerable way. As a consequence, cell growth slowed down and elongated buds appeared during cell division.

More recently, a novel kind of aptamer, indirectly activated by light, was realized by Lotz et al. [70]. This RNA structure binds molecules of azoCm, i.e., the trans photoisomer of azobenzene modified with chloramphenicol. Upon irradiation with 360-nm-wavelength light, azoCm falls into a configuration (the cis photoisomer) that is unable to bind the RNA aptamer. The RNA-binding trans photoisomer is re-established by exposing the cis photoisomer to light at 420-nm wavelength. Hence, light activation is conferred to the azoCm aptamer by the peculiar characteristics of its ligand.

After describing many synthetic aptamers, we shall mention the thiamine pyrophosphate (TPP) riboswitches, i.e., the only natural eukaryotic riboswitches discovered so far. They were found—usually in introns, as splicing regulators—in the genome of plants [71], filamentous fungi [72], algae, and marine phytoplankton [73]. Donovan et al. [74] showed that TPP riboswitches are present in some budding yeast species, such as *Candida parapsilosis*, and are functional in *S. cerevisiae*. Once placed on an intron inside a reporter protein gene, the TPP riboswitch allowed splicing and, therefore, fluorescence expression only in the absence of thiamine, whereas the pre-mature mRNA was not spliced in the presence of 10 µM thiamine.

An overview of the performance of the aptamers described in this section is given in Table 1.

## 3. CRISPR-Cas, Ribozymes, and Hairpins

So far, we have described RNA structures that are regulated by chemicals and represent possible interface between synthetic gene circuits and the environment. However, ribozymes and other hairpins that are inert to small molecules can also carry out important functions as components of genetic circuits. For instance, both HH and HDV ribozymes have been recently combined with the CRISPR-Cas9 system.

The type II clustered regularly interspaced short palindromic repeats—CRISPR-associated protein 9 (CRISPR-Cas9 system) has been widely adopted for gene editing and synthetic biology applications in eukaryotic cells [75]. Cas9 (or its nuclease-deficient version, termed dCas9) is brought to the DNA by a single guide RNA (sgRNA) molecule, which consists of a spacer sequence (complementary to the target DNA) and a hairpin-like structure, the direct repeat (overall, the sgRNA is ~100-nt long) [76]. The Cas9:sgRNA complex specifically binds the target DNA upstream of the protospacer adjacent motif (PAM—NGG or NAG) site and Cas9 generates a double-stranded DNA break 3 nucleotides upstream of PAM. The sgRNA, like natural short RNA sequences, is generally expressed in yeast and higher eukaryotes via RNA polymerase III-dependent elements such as the *SNR52* promoter and the *SUP4* terminator [77]. Alternatively, a ribozyme-gRNA-ribozyme (RGR) expression cassette, placed between the RNA polymerase II-type *ADH1* promoter and terminator, can be adopted as in [78,79]. In the work by Gao and Zhao [78], the sgRNA was flanked by an HHR [80] at the 5′-end and an HDV ribozyme [81] at the 3′-end. Upon transcription, the two ribozymes autocleaved and released the sgRNA that led Cas9 to cleave the target *GFP* gene with a dramatic reduction in the cell fluorescence level (see Figure 2d).

A similar strategy was followed by Gander et al. [82] to design CRISPR-dCas9-based NOR gates in *S. cerevisiae*. In this work, NOR gates consist of a constitutive synthetic promoter that is repressed by two different sgRNAs (input) and leads the transcription of an RGR cassette that releases a third sgRNA (output). The RGR was designed in silico to assure a correct folding of the ribozymes. Moreover, in order to increase repression efficiency, dCas9 was fused to the Mxi1 repression domain. NOR gates’ shared input–output allowed gates’ assembly into larger circuits such as an XOR gate and a six-step cascade.

A recent study showed that the fusion of a chemical-responsive aptamer to the sgRNA could lead, in bacteria, to ligand-activated/deactivated CRISPR-Cas9 systems [83]. However, this design—employing a theophylline aptamer—did not work in eukaryotes, including *S. cerevisiae*. A possible decrease in the affinity between the synthetic sgRNA and Cas9 has been proposed as an explanation of the negative result.

A different engineering approach [84] took advantage of the Csy4 protein (later renamed as Cas6f) that belongs to the type I-F CRISPR-Cas system from *Pseudomonas aeruginosa*. Csy4 processes the CRISPR array transcript into mature CRISPR-RNA (crRNA) after binding a 28-nt-long hairpin and cleaving the RNA at its base [85,86]. Qi et al. [84] inserted this hairpin between the start codon and the rest of the sequence of a fluorescent protein to test if the production of the reporter protein could be reduced by expressing Csy4. In *S. cerevisiae*, cleavage by Csy4 led to an effective gene silencing with an over ten-fold decrease in the fluorescent signal.

## 4. Hairpins Cleaved by RNase III

**Figure 3:**
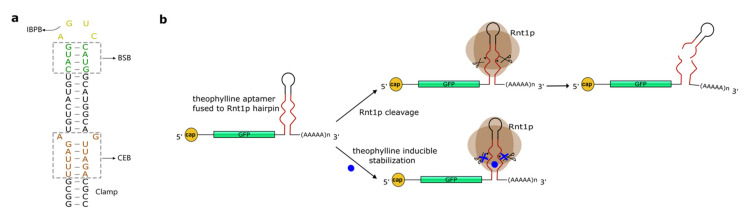
Rnt1p substrate. (**a**) The hairpin cleaved by the Rnt1 protein consisted of three main parts: The IBPB (initial binding and positioning box); the BSB (binding stability box); and the CEB (cleavage efficiency box). The “Clamp” is a spacer sequence needed to insulate and stabilize the hairpin [92]. (**b**) Theophylline aptamer fused to the Rnt1p hairpin substrate in the GFP 3′UTR. In the presence of theophylline, the secondary structure of the Rnt1p substrate is modified, which inhibits the cleavage by Rnt1p.

Csy4 is not an isolate case of a protein that induces RNA degradation upon recognition and cleavage of an RNA secondary structure. Another typical example is given by the RNase III endoribonucleases that discern and split double-stranded RNA (dsRNA) sequences. In this way, they induce the decay of various kinds of RNAs (e.g., small nuclear RNAs, ribosomal RNA precursors, small nucleolar RNAs, and messenger RNA) both in prokaryotic and eukaryotic cells [87]. *S. cerevisiae* RNase III (Rnt1protein—Rnt1p) targets RNA hairpins capped, generally, with the tetraloop AGNN [88] and cleaves them fourteen nucleotides upstream and sixteenth nucleotides downstream of the tetraloop [89,90]. More precisely, the Rnt1p substrate is divided into three regions: The initial binding and positioning box (IBPB), i.e., the tetraloop; the binding stability box (BSB), which consists of the four base-pairs adjacent to the tetraloop; and the cleavage efficiency box (CEB), which is a 6-nt-long part of the hairpin stem that contains the cleavage sites [87] (see Figure 3a).

In 2007, Lamontagne and Abou Elela [91] showed that Rnt1p could be used to degrade U2 snRNA (small nuclear RNA) in vivo by transforming yeast cells with synthetic guide RNAs that contained both a short hairpin capped with a tetraloop recognized by Rnt1p and an 11-nt-long sequence complementary to the U2 snRNA target. Due to the instability of the guide RNAs, U2 snRNA degradation could be detected only up to 30 min after cell transformation.

A few years later, Babiskin and Smolke [92] developed a library of 16 synthetic hairpins cleaved by Rnt1p.These novel synthetic RNA structures proved to be effective modules for regulating translation, in *S. cerevisiae*, upon placement in the 3′UTR of a target gene. The hairpins were designed by randomizing the 12 nucleotides inside the CEB and adding a four-G-C-pair clamp below the CEB itself, to achieve structural stability. Initially, the effect of the hairpins was tested on the expression of green and red fluorescence. The reduction in GFP synthesis varied from 7.9% to 84.7% of the construct without the hairpin. Subsequently, six of the hairpins in the library were placed downstream of the endogenous *ERG9* gene that encodes for squalene synthase, an enzyme employed, by yeast cells, in the metabolic pathway for the ergosterol production. The effects on *ERG9* showed a high correlation with those on the fluorescence protein genes. This work was later extended with the creation of another library of 16 hairpins obtained by randomizing the BSB (8 nt) [93]. Interestingly, this new (binding) library exhibited a smaller regulatory range (25% to 75%) with respect to the first (cleavage) library [94]. Therefore, the binding library appears more appropriate for fine-tuning of gene expression. Finally, Babiskin and Smolke [95] also built a series of Rnt1p switches by integrating theophylline aptamers into the CEB domain of Rnt1p hairpins. Upon binding of theophylline, the switch could no longer be cleaved by Rnt1p (ON switch—see Figure 3b). The performance of these new sensing devices was tuned by altering the aptamer (i.e., the CEB), the BSB, and the number of switches along the 3′UTR of the target (GFP) gene. The overall best configuration, obtained by using three switches, caused a 5.57-fold increase in fluorescence expression (two switches gave a maximal 4.21-fold gain; a single switch a 2.47-fold boost).

## 5. Others RNA Structures

Among the RNA structures which are of interest for the yeast synthetic biology community and do not belong to any of the above-described categories, it is worth mentioning the snorbozyme [96], i.e., a hybrid RNA molecule obtained from the fusion of a U3 small nucleolar RNA (snoRNA) with an HHR. The snorbozyme targeted other U3 snoRNA molecules with a 90% efficiency in vivo upon expression via multi-copy plasmids (i.e., the amount of the snorbozymes was about 10-fold higher than that of the target). If the two interacting molecules were present in equimolar amount in the cell nucleolus, the cleavage efficiency dropped to 60%.

A protein-responsive ribozyme, able to work both in *S. cerevisiae* and mammalian cells, was also realized as a hybrid construct by combing the MS2-coat-protein-binding aptamer [97] and the sTRSV (satellite RNA of tobacco ringspot virus) self-cleaving HHR [98]. The MS2 aptamer was attached either to stem I or II of the HHR, with the generation of two ON-genes configurations, where MS2 binding hindered ribozyme cleavage, and one OFF-gene mode, in which MS2 activated the HHR upon docking to its own aptamer. This RNA structure, named MS2-HHR, was placed in the 3′UTR downstream of the *GFP* gene. Fluorescence intensity increased from 20% to 97% of the maximal GFP level by using the ON-gene switch, whereas it decreased from 55% to 13% with the OFF-gene switch. Moreover, only the MS2 concentration was found to be an important parameter for the working of the two switches, i.e., MS2 localization in the nucleus or in the cytoplasm did not play a major role in the activity of the switches.

As a final work, we want to mention a recent study focused on the design of novel RNA hairpin modules that allow for fine-tuning in a predictive way and translation in *S. cerevisiae* [99]. The expression level of a fluorescent protein was modeled as a logistic function of the minimal folding energy (MFE—calculated via RNAfold [100]) of the hairpins that would be inserted into the 5′UTR of the reporter protein gene. This approach permitted to generate a library of hairpins that showed a broad range of protein synthesis downregulation (up to 100-fold).

## 6. Conclusions

Ribozymes and riboswitches have been discovered in bacteria and quickly repurposed in eukaryotic cells. In this review, we presented the progress made, mostly over the last two decades, in the constructions of artificial aptamers for applications into *S. cerevisiae* synthetic gene circuits. These RNA structures can be regulated, directly, with chemicals. Hence, they allow for a close control of protein synthesis to be achieved. Several synthetic ribozymes/riboswitches have proved their utility in the assembly of genetic networks, especially Boolean gates where a switch-like behavior is essential for a proper reproduction of a truth table. However, these RNA structures have, basically, never been tested into different kinds of even basic circuits (such as feedback loops and oscillators) and their general applicability to synthetic gene networks, in *S. cerevisiae*, is still to be confirmed.

If employed into digital circuits, riboswitches could drastically reduce the number of components necessary to mimic complex logic functions made of several literals (corresponding to the input chemicals) and clauses (the Boolean gates), as we have shown in a previous work [2]. Hence, the usage of highly effective aptamers can facilitate the construction of complex biosensors and bio-computing devices that, otherwise, would require the de novo construction of orthogonal transcription factor proteins. It should be noted, however, that the CRISPR-Cas technology can also be an efficient way to replace a high number of proteins with as many sgRNA molecules. In this context, the RGR cassette has proven to be a useful instrument for regulated sgRNA expression since it is based on RNA polymerase II-dependent DNA elements. As a consequence, synthetic or natural ribozymes could still find a place inside intricate logic networks.

Protein decay, moreover, can be achieved indirectly by inducing the degradation of the corresponding mRNA via a ribozyme or a Csy4-bound hairpin placed at the 3′UTR of a CDS. These represent simpler bio-engineering solutions than those so-far adopted, based on tagging proteins with a degron element recognized by the bacterial ClpP-ClpX system—repurposed in *S. cerevisiae* by Grilly et al. [101]—or coupled to the split-ubiquitin system [102].

Even though the working mechanisms of ribozymes and riboswitches look rather simple, engineering new ribozymes and riboswitches can be challenging. In order to carry out their function, these RNA molecules have to fold properly, once inserted along an mRNA sequence, and show high specificity towards a single chemical. Moreover, so far, not many chemical-aptamer systems have been characterized. Despite the fact that, over the past years, new ribozymes and riboswitches have been engineered in mammalian cells [103,104,105], their portability to yeast appears difficult since the working of, for instance, HHRs has been shown to be, generally, highly context dependent [106]. This is a relevant limitation compared to the use of foreign (i.e., orthogonal) proteins as wiring agents between circuit components since even bacterial proteins have shown to be active in yeast. Therefore, only innovative screening methods such as Capture-SELEX can accelerate the discovery of new inducible ribozyme/riboswitches able to work in *S. cerevisiae*.

Notwithstanding the non-negligible hurdles that currently hinder a diffuse usage of aptamer-like structures in genetic circuits, we think that the application of ribozymes to the CRISPR-Cas technology for the expression of sgRNAs and the ongoing studies about using hairpins to build chemical-responsive crRNAs are a clear indication that the engineering of novel synthetic ribozymes/riboswitches will play a central role in synthetic biology in the coming years.

## Figures and Tables

**Table 1:** Aptamer performance overview. The effect on gene expression due to the chemical-responding aptamers described in this work are here summarized.

RNA Structure	K_d_	Additional RNA	Action Triggered by the Chemicals	Performance	Reference(s)
Theophylline aptamer mTCT8-4	0.1 μM	Short antisense RNA	Increase/decrease in gene expression upon binding near the START codon	90% reduction (OFF switch) or increase (ON switch) in fluorescence expression (1 to 10 mM of theophylline)	[32]
		HHR	Induction/inhibition of ribozyme self-cleavage	14-fold increase in fluorescence expression from an AND gate (5–10 mM theophylline and 0.25–0.5 mM tetracycline)	[33,34]
		-	Translational frame shift (-1 PRF)	7.0-fold increase or 5.9-fold decrease in the translation frame shift (40 mM theophylline)	[38]
Tetracycline aptamer	0.8 nM	-	Translation inhibition upon placement on the 5’ UTR of *gfp*	Single aptamer: 6-to 9-fold fluorescence repression; 2 and 3 aptamers: 21-fold and 37-fold fluorescence repression, respectively. In every case, 250 μM tetracycline were used	[44,45]
		Intron	Pre-mRNA splicing	Unquantified fluorescence reduction	[47]
		HHR	Ribozyme self-cleavage	Complete self-cleaved (1 μM tetracycline)	[48]
Neomycin aptamer	-	-	Translational frame shift (-1 PRF)	5.0-fold enhancement or 4.2-fold reduction in the translation frame shift (550 µM neomycin)	[37]
Neomycin aptamer N1 Neomycin aptamer N1-based riboswitch	- -	*S. mansoni* HHR	Translation inhibition upon insertion on the 3’UTR of *gal4*	Around 25-fold *lacZ* expression downregulation (100 μg/mL)	[60,61]
		Inactive env-9 twister ribozyme	Translation inhibition upon insertion on the 3’UTR of *gal4*	About 10-fold decrease in *lacZ* expression	[62]
Neomycin aptamer M4 or M7	-	*S. mansoni* HRR	Translation inhibition upon insertion on the 3’UTR of *gfp*	Around 2-fold fluorescence upregulation (ON switch) and 3-fold fluorescence downregulation (OFF switch) (100 μg/mL neomycin)	[64]
Neomycin aptamer M4	-	-	Translation inhibition	7.5-fold fluorescence repression (100 µM neomycin)	[54]
PARO riboswitch (paromomycin)	20 nM	-	Translation inhibition	8.5-fold decrease in gene expression (250 μM paromomycin)	[66,67]
				13.8-fold decrease in gene expression from a NOR gate (250 μM of both paromomycin and neomycin)	
fluoroquinolone ciprofloxacin riboswitch	64.2 nM	-	Translation inhibition	7.5-fold fluorescence downregulation	[68]
Tetra- methylrosamine aptamer	-	-	Translation inhibition upon placement on the 5’ UTR of *CLB2*	Reduction in cell growth	[69]
azoCm aptamer	-	-	Configurational change	Unquantified control of gene expression	[70]
TPP (thiamine pyrophosphate) riboswitch	-	Intron	Splicing inhibition	pre-mRNA is not spliced in the presence of 10 µM thiamine	[74]
